# Ethanol Extract of Artemisia Annua Prevents LPS-Induced Inflammation and Blood–Milk Barrier Disruption in Bovine Mammary Epithelial Cells

**DOI:** 10.3390/ani12101228

**Published:** 2022-05-10

**Authors:** Jie Song, Yao Hu, Lifang Wang, Changjin Ao

**Affiliations:** 1 Inner Mongolia Key Laboratory of Animal Nutrition and Feed Science, College of Animal Science, Inner Mongolia Agricultural University, Hohhot 010018, China; songjiesau@163.com (J.S.); 18447055727@163.com (Y.H.); 2 Laboratory of Quality and Safety Risk Assessment for Agricultural Products (Hohhot), Ministry of Agriculture and Rural Affairs, Inner Mongolia Academy of Agricultural and Animal Husbandry Sciences, Hohhot 010031, China

**Keywords:** Artemisia annua extract, lipopolysaccharide, inflammation, CD36, tight junction proteins

## Abstract

**Simple Summary:**

Mastitis is one of the most serious diseases restricting the development of the dairy industry. It not only affects bovine production performance, but also leads to antibiotic residues and drug resistance. As antibiotics have gradually been forbidden, it is urgently necessary to find natural alternatives to prevent mastitis. Artemisia annua is an annual low-toxicity herb native to China that is widely distributed around the world. This study aimed to explore whether an ethanol extract of Artemisia annua (AAE) could be used as a new preventive agent to ameliorate LPS-induced inflammation and blood–milk barrier disruption in bovine mammary epithelial cells (bMECs). Our results showed that AAE pretreatment could alleviate inflammatory injury and tight junction abnormalities after LPS challenge, which was partially related to the attenuation of nuclear factor-κB (NF-κB) signaling and the downregulation of CD36 levels. Our study can provide a certain reference value for the development and utilization of plant extracts.

**Abstract:**

This experiment evaluated the pre-protective effect of AAE on inflammatory injury and tight junction disturbance in bMECs induced by LPS. The bMECs were treated with AAE (3, 6, 12 μg/mL) for 3 h and then incubated with 10 μg/mL lipopolysaccharide (LPS) for 12 h. Our results showed that LPS significantly increased the mRNA and protein expression of CD36, induced the phosphorylation of IκBα and p65 and elevated the levels of TNF-α, IL-1β and IL-6 mRNA, which further resulted in ultrastructural damage, disrupted the expression of tight junction proteins (occludin, zonula occludens (ZO-1) and claudin-1) and decreased the viability of bMECs (*p* < 0.05). More importantly, AAE pretreatment attenuated the expression of CD36, suppressed the activity of the NF-κB signaling pathway and down-regulated the levels of inflammatory factors in LPS-stimulated bMECs (*p* < 0.05). Therefore, AAE can effectively protect bMECs against inflammatory injury and tight junction dysfunction, which has important research value for the prevention of bovine mastitis.

## 1. Introduction

Mastitis is recognized as one of the most common bovine diseases worldwide [1]. This disease can directly lead to inflammatory damage to mammary tissues, alter the integrity of the blood–milk barrier and impair the secretory function of bovine mammary epithelial cells (bMECs) [2,3,4]. A variety of pathogenic microorganisms can induce mastitis, and Gram-negative Escherichia coli (*E. coli*) infection is one of the main reasons [5]. Lipopolysaccharide (LPS), an important virulence factor in the cell wall of *E. coli*, is considered to be a major pathogen-associated molecular pattern (PAMP) that causes acute bovine mastitis with a usually fast recovery rate [6,7].

Cluster of differentiation 36 (CD36) acts as a fatty acid-binding protein, carrying long-chain fatty acids (LCFAs) through the capillary endothelium into mammary epithelial cells [8]. In addition, CD36 also plays a critical role in natural immunity and can be regarded as a pattern recognition receptor (PRR) that identifies pathogenic microorganisms and participates in the inflammatory response of cells [9]. Nuclear factor-κB (NF-κB) is a well-known regulator of the inflammatory response [10]. Recent studies have shown that CD36, Toll-like receptor 4 (TLR4) and TLR6 form heterotrimeric complexes after PAMP recognition, further triggering NF-κB signaling and producing a series of proinflammatory mediators [11]. Surprisingly, CD36 can even activate c-Jun N-terminal kinase 1/2 (JNK1/2) and IL-8 secretion in LPS- or *E. coli*-exposed cells through TLR4-independent pathways [12]. In dairy goat mammary epithelial cells, CD36 can mediate downstream NF-κB pathways and activator protein 1 (AP-1) after LPS treatment, which induces the secretion of tumor necrosis factor α (TNF-α) and interleukin 1β (IL-1β) [13]. The tight junction (TJ) is the main structure of the blood–milk barrier, and reduced tight junction proteins (TJP) can aggravate intracellular inflammation [14]. Studies on a rat obesity model have shown that the downregulation of the CD36 mRNA level is related to a decrease in TNF-α and an increase in intestinal TJP (occludin) mRNA expression [15]. Therefore, CD36 may be a valuable therapeutic target for bovine mastitis.

Currently, antibiotics are the first choice to treat bovine mastitis, but long-term abuse of antibiotics may lead to pathogen resistance and antibiotic residues, which seriously endanger human health [16,17]. Thus, there is an urgent need for new therapies to prevent and treat mastitis. Artemisia annua (*A. annua*), a species of the genus Artemisia in the Compositae family, is an annual low-toxicity herb native to China that is widely distributed around the world. *A. annua* extracts have received extensive attention due to their remarkable antibacterial [18], anti-inflammatory [19], immunoregulatory [20] and anti-oxidative effects [21]. Reports have shown that *A. annua* extracts has anti-inflammatory effects and can suppress PGE2, NO, TNF-α and IL-6 expression and reduce IκB phosphorylation levels in RAW264.7 macrophages stimulated with LPS [22]. However, the anti-inflammatory mechanism of *A. annua* extracts on the LPS-stimulated inflammatory response in bMECs remains unclear. Therefore, we evaluated the protective mechanism of *A. annua* (AAE) against inflammatory injury and tight junction dysfunction in bMECs stimulated with LPS.

## 2. Materials and Methods

### 2.1. Preparation and Component Identification of AAE

AAE freeze-dried powder was derived from the whole plant of *A. annua* and produced by Zelang Biotechnology (Nanjing, China). The main ethanol extraction process has been listed in our previous articles [23]. The active components of AAE were analyzed by UPLC (Nexera X2, Shimadzu company, Tokyo, Japan) and MS/MS (4500QTRAP, Applied Biosystems, Framingham, MA, USA). The AAE pre-processing method was based on Wang et al. [24]. The chromatographic and mass spectrometry conditions were based on Duan et al. [25]. In short, chromatographic conditions were as follows: chromatographic column (181.8 µm, 2.1 mm × 100 mm, Agilent); mobile phase—phase A was ultrapure water (add 0.1% formic acid), phase B was acetonitrile (add 0.1% formic acid); flow rate—0.35 mL/min; elution gradient (water/acetonitrile)—95:5 (*v*/*v*) for 0 min, 5:95 (*v*/*v*) for 9.0 min, 5:95 (*v*/*v*) for 10.0 min, 95:5 (*v*/*v*) for 11.1 min and 95:5 (*v*/*v*) for 14.0 min; the column temperature was 40 °C and the injection volume was 4 µL. The mass spectrum conditions were as follows: the temperature of the electrospray ionization source was 550 °C; mass spectral voltage, positive ion mode (5500 V)/negative ion mode (−4500 V); curtain gas (CUR) was 25 psi, and the collision activated dissociation (CAD) parameter was high. In QQQ, each ion pair is scanned and detected according to the optimized clustering potential (DP) and collision energy (CE). The active components in AAE were analyzed qualitatively based on the MWDB database (Metware Biotechnology Co., Ltd., Wuhan, China).

### 2.2. Cell Culture and Treatments

Mammary epithelial cells were derived from mixed mammary tissue samples of 3 healthy lactating Holstein cows without mastitis symptoms (milk somatic cell counts < 10^5^ cells/mL). The isolation and purification methods of bMECs were based on Dan et al. [26] and Dai et al. [27]. In short, the mammary tissues were collected aseptically, washed in PBS buffer containing 1% penicillin–streptomycin mixture and cut into pieces by sterile ophthalmic scissors. Then, type II collagenase (Gibco, Grand Island, NY, USA) was used to digest the minced mammary tissue in a 5% CO_2_ incubator at 37 °C for 1 h; the bMECs and tissue blocks were separated by an 80-mesh filter. Finally, cells were collected by centrifugation (1500 rpm, 3min) and cultured at 37 °C in 5% CO_2_. The cell culture medium consisted of DMEM/F12 (Gibco, Grand Island, NY, USA) supplemented with 10% fetal bovine serum (Bioind, Kibbuiz, Israel), 1% penicillin–streptomycin mixture (Gibco, Grand Island, NY, USA), 2.5 μg/mL amphotericin B (Coolaber, Beijing, China), 1 μg/mL hydrocortisone (Sigma–Aldrich, St Louis, MO, USA), 10 ng/mL epidermal growth factor (Sigma–Aldrich, St Louis, MO, USA) and insulin transferrin selenium (Gibco, Grand Island, NY, USA). Cells at passages 3 to 4 were selected for the experiment. AAE and LPS (*E. coli* serotype O111:B4, Sigma, St Louis, MO, USA) were dissolved in DMEM/F12 without serum and antibiotics. All cells were starved in serum-free DMEM/F12 for 24 h before any treatment. There were 5 treatments in total. CON: cells were routinely cultured in medium (serum-free) for 15 h. LPS: cells were cultured in medium (serum-free) for 3 h, washed twice with PBS and then incubated with 10 μg/mL LPS for 12 h. AAE-3, AAE-6 and AAE-12: cells were treated with 3, 6 and 12 μg/mL AAE, respectively, for 3 h before being washed and incubated with 10 μg/mL LPS for 12 h.

### 2.3. Immunofluorescence Analysis

In order to identify the isolated and purified bMECs, the expression of cytokeratin 18 (CK-18) was detected by immunofluorescence. The cell slides were fixed on ice with 4% paraformaldehyde for 15 min and washed twice with precooling PBS for 5 min each time. After blocking at 37 °C for 1 h with 5% normal goat serum in PBS, the cells were treated with rabbit anti-keratin 18 antibody (1:100 dilution, Sigma-Aldrich, St Louis, MO, USA) overnight at 4 °C and washed with PBS 5 times. Subsequently, the cells were incubated at 37 °C for 1 h in the dark with the FITC labeled goat anti-rabbit IgG (1:200 dilution, Medical Discovery Leader (MDL, Beijing, China) and washed with PBS again 3 times. Then, the cells were stained with DAPI in the dark for 2 min. Finally, the cells were sealed with glycerol and observed immediately under a fluorescence microscope (Leica DM3000, Leica Microsystems, Wetzlar, Germany).

### 2.4. Cell Viability

The 3-(4,5-dimethylthiazol-2-yl)-2,5-diphenyltetrazolium (MTT) method was used to measure cell viability according to Liu et al. (2013). Cells were cultured at a density of 1.5 × 10^4^ cells/well in 96-well plates. To study the effect of AAE on cell viability without LPS, the bMECs were treated with 3, 6, 12, 24 and 48 μg/mL AAE for 3, 6, 12, 24 and 48 h. To study the protective effect of AAE on the cell viability of LPS-induced bMECs, AAE (3, 6 and 12 μg/mL) or 10 μg/mL LPS was added for different times. Then, 10 μL MTT (5 mg/mL) was added (4 h). After the supernatant was removed, 100 μL/well dimethyl sulfoxide (DMSO) was added and oscillated for 10 min. The absorbance (490 nm) was measured by an automatic microplate reader (Synergy H1, Biotek Instruments, Inc., Winooski, VT, USA).

### 2.5. Flow Cytometry

Cells (1.5 × 10^6^ cells/well) in 6-well plates were treated with LPS (10 μg/mL) or AAE (3, 6 and 12 μg/mL) and detached with trypsin. Apoptosis was examined using an annexin V-FITC apoptosis detection kit (Beyotime, Shanghai, China) according to the instructions. Briefly, the cell suspension was centrifuged at 1000 g for 5 min. After adding 195 μL annexin V-FITC binding solution, cells were stained with 5 μL annexin V-FITC and 10 μL propidium iodide dyeing solution (PI), incubated at room temperature (20–25 °C) for 10-20 min and then analyzed immediately by flow cytometry (Beckman Coulter Cytoflex S, Krefeld, Germany).

### 2.6. Transmission Electron Microscopy

Cell samples (1.5 × 10^6^ cells/well) were collected and washed with PBS. The samples were placed in 2.5% glutaraldehyde buffer, fixed overnight at 4 °C, washed 3 times with buffer and postfixed in 1% OsO_4_ at 4 °C for 2 h. After being washed, the cell samples were dehydrated with gradient alcohol and acetone for 10 min each and then infiltrated with epoxy resin acetone. After resin embedding, polymerization and slicing, ultrathin slices were placed on double copper mesh, stained with 1% uranium acetate for 30 min and 3% lead citrate for 5 min, dried and observed under a transmission electron microscope (TEM, FEI Tecnal G2F30, FEI Co., Hillsboro, OR, USA).

### 2.7. Quantitative Real-Time PCR

According to the manufacturer’s instructions, TRNzol Universal Reagent (Tiangen, Beijing, China) was used to extract total RNA from bMECs (10^6^ cells/well). Reverse transcription reactions were carried out with a FastKing gDNA Dispelling RT SuperMix kit (Tiangen, Beijing, China). Quantitative real-time PCR (qRT-PCR) was performed using SuperReal PreMix Plus (SYBR Green; Tiangen, Beijing, China) with a LightCycler 480II fluorescent quantitative PCR instrument (Roche, Penzberg, Germany). According to the research of Andersen et al. (2004), UXT was determined as the internal reference gene with the best stability among different treatments by NormFinder software in this study. The primer sequences are shown in Table 1. The relative gene expression was calculated by the 2^−ΔΔCT^ method.

### 2.8. Western Blotting Analysis

Cells (10^6^ cells/well) were sonicated in an ice bath, lysed with lysis solution containing protease inhibitors and then centrifuged at 4 °C and 12,000 rpm for 15 min. The total protein concentration was determined by a BCA protein concentration assay kit (MD913053; MDL, Beijing, China). The proteins were separated by SDS–PAGE (10%) and transferred onto polyvinylidene difluoride (PVDF) membranes (ISEQ00010, 0.22 μm; Millipore, Billerica, MA, USA) by transfer electrophoresis. The membrane was blocked with nonfat dry milk (5%) in Tris-buffered saline–Tween (TBST) at room temperature for 1 h. The proteins on the membrane were reacted with the corresponding primary antibodies against IκBα, phospho-IκBα, p65, phospho-p65, CD36 (bs-1287R, bs-2513R, bs-0465R, bs-0982R, bs-1100R; Bioss, Beijing, China) and β-actin (MD6553; MDL, Beijing, China) overnight at 4 °C. The membrane was incubated with the anti-rabbit secondary antibodies (1:4000; MD912577; MDL, Beijing, China) at room temperature for 1 h in the dark after being washed with TBST. The membrane was washed with TBST again, and finally, the protein bands were captured with a chemiluminescence imaging system (ChemiScope 6100, CLINX Science Instruments, Shanghai, China).

### 2.9. Statistical Analysis

The data are expressed as the mean ± standard error of the mean (mean ± SEM) and were analyzed by SPSS 20.0 software (SPSS Inc.). One-way ANOVA and Duncan’s test were used to compare the data among groups. *p* < 0.05 and *p* < 0.01 indicated a significant and an extremely significant difference, respectively. The data were collected at least in triplicate.

## 3. Results

### 3.1. Analysis of Active Components in AAE

The active components of AAE were analyzed by UPLC-MS/MS. In this study, 321 compounds were identified in AAE, including 57 phenolic acids, 48 flavonoids, 39 organic acids, 18 alkaloids, 16 terpenoids, 12 lignans and coumarins, 2 quinones and 129 others. The 30 representative active components in AAE are listed in Table 2.

### 3.2. Immunofluorescence Identification of CK-18 in bMECs

**Figure 1:**
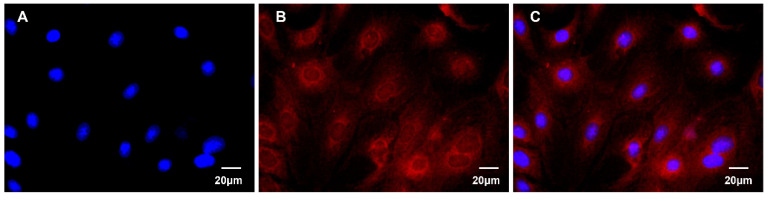
Immunofluorescence identification of cytokeratin 18 (CK-18) in bovine mammary epithelial cells (bMECs). (**A**) The nuclei were stained by DAPI. (**B**) The CK-18 was labeled with FITC. (**C**) The merged micrographs showed that CK-18 existed in the cytoplasm of bMECs.

Cytokeratin is an epithelial cell-specific cytoskeleton. The expression of CK-18 in bMECs was detected by immunofluorescence. The results showed that the expression of CK-18 in the isolated and purified cells was positive. The nuclei were stained by DAPI (Figure 1A). The CK-18 existed in the cytoplasm of the cells and was labeled with FITC (Figure 1B,C). Therefore, it was illustrated that the cells were bMECs.

### 3.3. AAE Increased Cell Viability of bMECs

**Figure 2:**
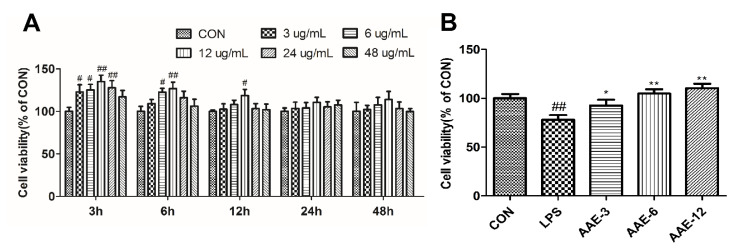
Ethanol extract of Artemisia annua (AAE) increased cell viability of bMECs. (**A**) The bMECs were treated with different concentrations of AAE (3, 6, 12, 24 and 48 μg/mL) for 3, 6, 12, 24 and 48 h. (**B**) The bMECs were treated with different concentrations (3, 6 and 12 μg/mL) of AAE for 3 h, and then incubated with 10 μg/mL LPS for 12 h. Cell viability was determined by MTT method. CON: cells were cultured in medium (serum-free) for 15 h; LPS: cells were cultured in medium (serum-free) for 3 h and then incubated with 10 μg/mL LPS for 12 h; AAE-3, AAE-6 and AAE-12: cells were treated with 3, 6 and 12 μg/mL AAE for 3 h before 10 μg/mL LPS incubation for 12 h. Comparisons among groups were made with ANOVA followed by Duncan’s test. The data are expressed as mean ± SEM (*n* = 6). ^#^ *p* < 0.05 and ^##^ *p* < 0.01 vs. CON. * *p* < 0.05 and ** *p* < 0.01 vs. LPS.

The promoting effect of AAE on the viability of bMECs is shown in Figure 2. In this study, the addition time and dosage of AAE were selected by cell viability analysis. The bMECs were treated with different concentrations of AAE (3, 6, 12, 24 and 48 μg/mL) for 3, 6, 12, 24 and 48 h (Figure 2A). In general, 3–48 ug/mL AAE had no inhibitory effect on cell viability from 3 to 48 h. Treatment with 3, 6 and 12 ug/mL AAE for 3 h significantly promoted cell viability in a dose-dependent manner compared with CON (*p <* 0.05); however, 24 and 48 ug/mL AAE decreased the cell viability slightly. Based on the above results, pre-treatment with 3, 6 and 12 ug/mL AAE for 3 h was selected for the study of the preventive effect of AAE on LPS-induced bMEC. Then, compared with the CON group, 10 μg/mL LPS significantly decreased the viability of bMECs (*p* = 0.004, Figure 2B). However, 3, 6 and 12 μg/mL AAE had significant protective effects on the viability of bMECs challenged with LPS (*p* = 0.043, *p* = 0.001 and *p* < 0.0001, respectively).

### 3.4. AAE Protected bMEC from Apoptosis in Response to LPS Stimulation

**Figure 3:**
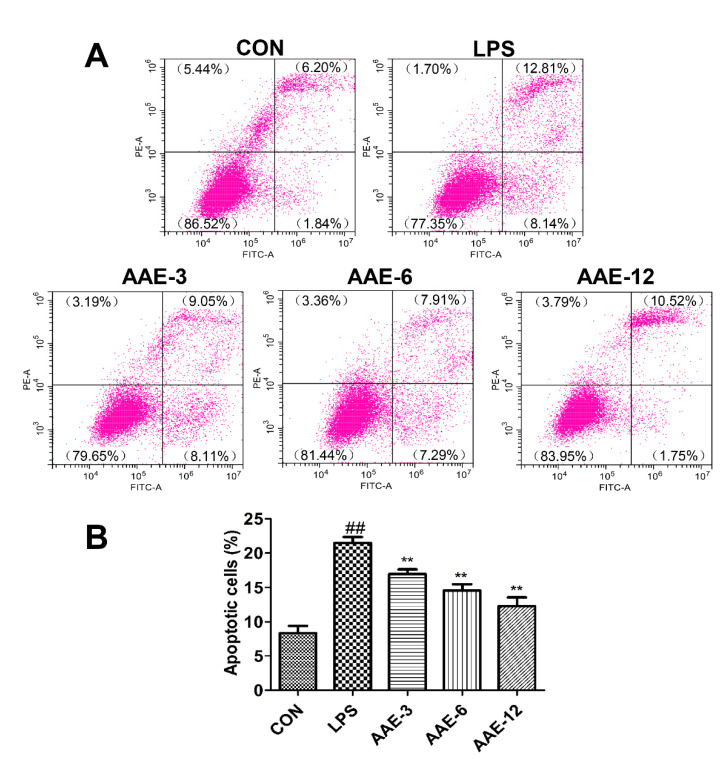
AAE prevented LPS-induced apoptosis. The bMECs were treated in the absence or presence of AAE (3, 6, 12 μg/mL) for 3 h, and then challenged by 10 μg/mL LPS for 12 h. (**A**) The percentage of apoptotic cells was detected by flow cytometer. (**B**) Early apoptotic cells and late apoptotic cells (the right two quadrants of each figure in Part A) were added together to compare the percentage of apoptotic cells. CON: cells were cultured in medium (serum-free) for 15 h; LPS: cells were cultured in medium (serum-free) for 3 h and then incubated with 10 μg/mL LPS for 12 h; AAE-3, AAE-6 and AAE-12: cells were treated with 3, 6 and 12 μg/mL AAE for 3 h before 10 μg/mL LPS incubation for 12 h. Comparisons among groups were made with ANOVA followed by Duncan’s test. The data are expressed as mean ± SEM (*n* = 3). ^##^ *p* < 0.01 vs. CON. ** *p* < 0.01 vs. LPS.

Apoptotic cells were examined by flow cytometry. In this study, 10 μg/mL LPS significantly induced apoptosis compared with that in the CON group (*p* < 0.0001). In contrast, the groups that were treated with 3 μg/mL (*p* = 0.008), 6 μg/mL (*p* = 0.001) and 12 μg/mL (*p* < 0.0001) AAE before LPS stimulation exhibited significantly reduced apoptosis (Figure 3).

### 3.5. Effect of AAE on Ultrastructural Changes

**Figure 4:**
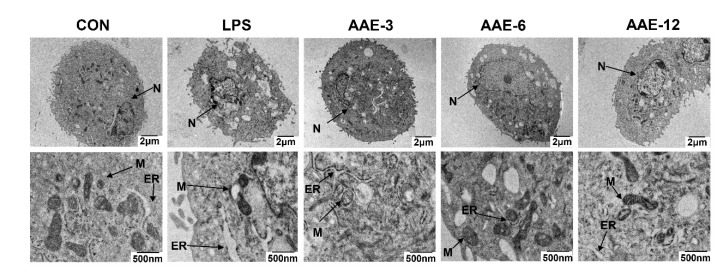
Effect of AAE on ultrastructural changes. The bMECs were treated with AAE (3, 6, 12 μg/mL) for 3 h, followed by 10 μg/mL LPS stimulation for 12 h. Transmission electron microscope (TEM) was used to test the protective effect of AAE on the ultrastructural changes of bMECs damaged by LPS. N, M and ER represent nucleus, mitochondria and endoplasmic reticulum, respectively. CON: cells were cultured in medium (serum-free) for 15 h; LPS: cells were cultured in medium (serum-free) for 3 h and then incubated with 10 μg/mL LPS for 12 h; AAE-3, AAE-6 and AAE-12: cells were treated with 3, 6 and 12 μg/mL AAE for 3 h before 10 μg/mL LPS incubation for 12 h.

The TEM results are shown in Figure 4. The ultrastructure of bMECs in the CON group was normal, without morphological damage, exhibiting complete cell membranes with slender microvilli, oval nuclei, normal endoplasmic reticula and abundant mitochondria. However, LPS stimulation elicited the breakdown of the microvilli, incomplete cell membranes, fragmented nuclei, expanded endoplasmic reticula, the disappearance of mitochondrial cristae and the vacuolation of cells. In contrast, AAE pretreatment had a marked protective effect on the ultrastructure of bMECs stimulated with LPS, and the high dose of AAE (12 μg/mL) exhibited the best effect.

### 3.6. Effect of AAE on the Expression of TJP in bMECs Injured by LPS

**Figure 5:**
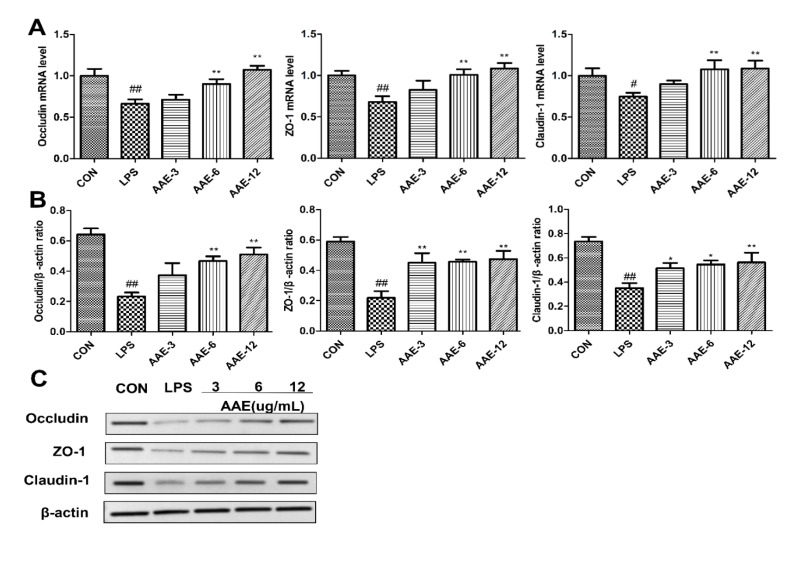
Effect of AAE on the expression of tight junction proteins in LPS-exposed bMECs. Cells were pretreated with three different concentrations (3, 6, 12 μg/mL) of AAE for 3 h before incubation with 10 μg/mL LPS for 12h. (**A**) Relative mRNA expression levels of occludin, ZO-1 and claudin-1 were measured by quantitative real-time PCR (qRT-PCR) (*n* = 6). (**B**) Relative protein expression levels of occludin, ZO-1 and claudin-1 to β-actin (*n* = 3). (**C**) Western blotting analysis was performed to test occludin, ZO-1 and claudin-1 protein levels; β-actin was used as endogenous control. The complete Western blotting images are shown in Figure S1. CON: cells were cultured in medium (serum-free) for 15 h; LPS: cells were cultured in medium (serum-free) for 3 h and then incubated with 10 μg/mL LPS for 12 h; AAE-3, AAE-6 and AAE-12: cells were treated with 3, 6 and 12 μg/mL AAE for 3 h before 10 μg/mL LPS incubation for 12 h. Comparisons among groups were made with ANOVA followed by Duncan’s test. The data are expressed as mean ± SEM. ^#^ *p* < 0.05 and ^##^ *p* < 0.01 vs. CON. * *p* < 0.05 and ** *p* < 0.01 vs. LPS.

The expression of three important TJPs (occludin, ZO-1, claudin-1) was detected by qRT-PCR and Western blotting. LPS significantly decreased the mRNA and protein expression levels of occludin (*p* = 0.001, *p* < 0.0001), ZO-1 (*p* = 0.007, *p* < 0.0001) and claudin-1 (*p* = 0.039, *p* < 0.0001). Compared with the LPS group, 3 μg/mL AAE enhanced the protein expression of ZO-1 (*p* = 0.005) and claudin-1 (*p* = 0.045); 6 and 12 μg/mL AAE pretreatment significantly improved the gene and protein expression of the three TJPs (*p* < 0.05) (Figure 5).

### 3.7. AAE Alleviated Inflammatory Cytokine Expression Stimulated by LPS

**Figure 6:**
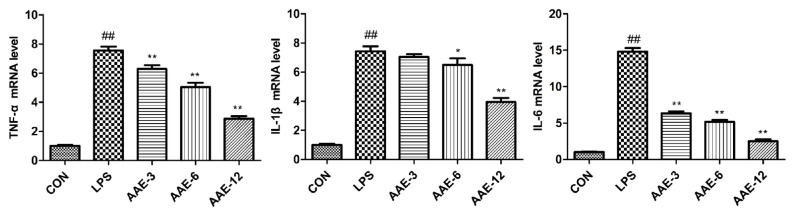
AAE downregulated the transcription of tumor necrosis factor α (TNF-α), interleukin-1β (IL-1β) and IL-6 gene expression in bMECs motivated by LPS. Cells were pretreated with three different concentrations (3, 6, 12 μg/mL) of AAE or medium without serum for 3 h and then treated with 10 μg/mL LPS for 12 h. Relative mRNA expression levels of TNF-α, IL-1β and IL-6 were measured by quantitative real-time PCR (qRT-PCR). CON: cells were cultured in medium (serum-free) for 15 h; LPS: cells were cultured in medium (serum-free) for 3 h and then incubated with 10 μg/mL LPS for 12 h; AAE-3, AAE-6 and AAE-12: cells were treated with 3, 6 and 12 μg/mL AAE for 3 h before 10 μg/mL LPS incubation for 12 h. Comparisons among groups were made with ANOVA followed by Duncan’s test. The data are expressed as mean ± SEM (*n* = 6). ^##^ *p* < 0.01 vs. CON. * *p* < 0.05 and ** *p* < 0.01 vs. LPS.

Inflammatory cytokines play an important role in regulating the inflammatory response. In this study, LPS significantly promoted the mRNA expression of inflammatory cytokines, such as TNF-α, IL-1β and IL-6, compared with that in the CON group (*p* < 0.0001) (Figure 6). AAE alleviated the inflammatory response and downregulated the mRNA levels of inflammatory cytokines in a dose-dependent manner. Among them, 12 μg/mL AAE significantly decreased the mRNA expression of TNF-α, IL-1β and IL-6 and showed the most significant anti-inflammatory effect (*p* < 0.0001).

### 3.8. AAE Attenuated the Levels of CD36 in bMECs Challenged with LPS

**Figure 7:**
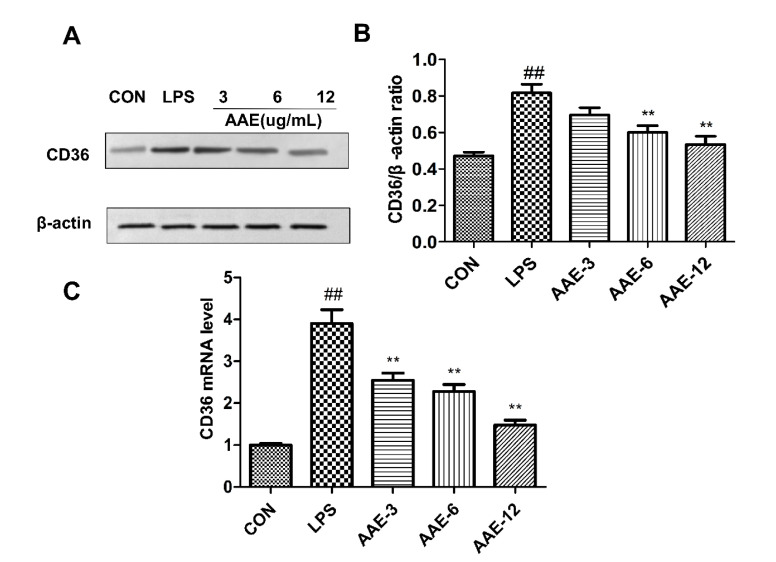
AAE reduced the expression of CD36 in bMECs induced by LPS. Cells were treated with various concentrations (3, 6, 12 μg/mL) of AAE or medium without serum for 3 h before stimulation with 10 μg/mL LPS for 12 h. (**A**) Western blotting analysis was performed to test CD36 protein level; β-actin was used as endogenous control. The complete Western blotting images are shown in Figure S2. (**B**) Relative protein expression levels of CD36 to β-actin (*n* = 3). (**C**) Relative mRNA expression levels of CD36 were measured by quantitative real-time PCR (*n* = 6). CON: cells were cultured in medium (serum-free) for 15 h; LPS: cells were cultured in medium (serum-free) for 3 h and then incubated with 10 μg/mL LPS for 12 h; AAE-3, AAE-6 and AAE-12: cells were treated with 3, 6 and 12 μg/mL AAE for 3 h before 10 μg/mL LPS incubation for 12 h. Comparisons among groups were made with ANOVA followed by Duncan’s test. The data are expressed as mean ± SEM. ^##^ *p* < 0.01 vs. CON. ** *p* < 0.01 vs. LPS.

To investigate whether CD36 participated in the protective effect of AAE against LPS-induced inflammation, qRT-PCR and Western blotting were used to analyze the gene and protein expression levels of CD36. The mRNA and protein expression levels of CD36 were significantly higher in the LPS group than CON (*p* < 0.0001) (Figure 7). However, pretreatment with different concentrations of AAE significantly inhibited CD36 mRNA expression levels compared with the LPS group (*p* < 0.0001) (Figure 7C). The effect of AAE on the protein expression of CD36 in LPS-challenged bMECs also showed a similar trend; 6 and 12 μg/mL AAE significantly inhibited the protein expression of CD36 in LPS-stimulated bMECs (*p* = 0.0003, *p* < 0.0001) (Figure 7A,B).

### 3.9. AAE Pretreatment Suppressed the LPS-Induced Activity of NF-κB Pathway

**Figure 8:**
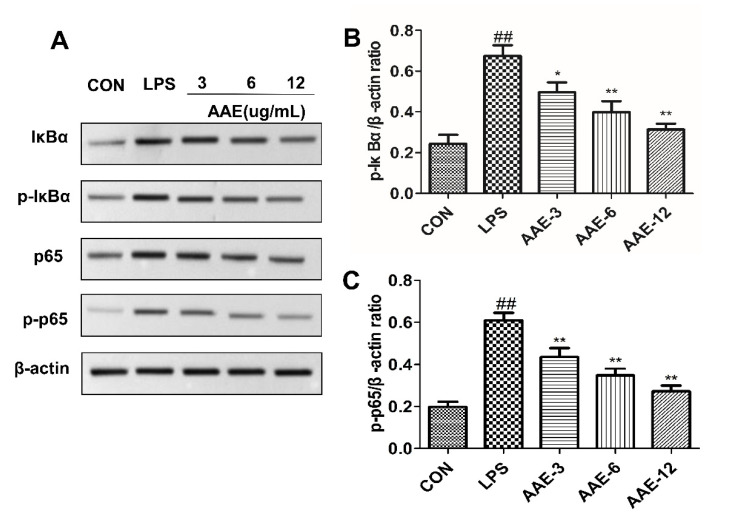
AAE pretreatment suppressed the LPS-induced activity of the NF-κB pathway. The bMECs were treated in the absence or presence of AAE (3, 6, 12 μg/mL) for 3 h, and then treated with 10 μg/mL LPS for 12 h. (**A**) Western blotting analysis was performed to test IκBα, p-IκBα, p65 and p-p65 protein levels; β-actin was used as endogenous control. The complete Western blotting images are shown in Figure S3. (**B**) Relative protein expression levels of p-IκBα to β-actin. (**C**) Relative protein expression levels of p-p65 to β-actin. CON: cells were cultured in medium (serum-free) for 15 h; LPS: cells were cultured in medium (serum-free) for 3 h and then incubated with 10 μg/mL LPS for 12 h; AAE-3, AAE-6 and AAE-12: cells were treated with 3, 6 and 12 μg/mL AAE for 3 h before 10 μg/mL LPS incubation for 12 h. Comparisons among groups were made with ANOVA followed by Duncan’s test. The data are expressed as mean ± SEM (*n* = 3). ^##^ *p* < 0.01 vs. CON. * *p* < 0.05 and ** *p* < 0.01 vs. LPS.

The NF-κB pathway is essential in inflammatory signal transduction. Therefore, Western blotting was used to investigate the phosphorylation levels of IκBα and p65 (Figure 8). Compared with those in the CON group, LPS sharply increased the IκBα and p65 phosphorylation levels, indicating the activation of the NF-κB signaling pathway (*p* < 0.0001). Pretreatment with 3, 6 and 12 μg/mL AAE showed significant anti-inflammatory effects and inhibited the transduction of the NF-κB signaling pathway effectively (p-IκBα: *p* = 0.023, *p* = 0.002 or *p* < 0.0001; p-p65: *p* = 0.004, *p* < 0.0001 or *p* < 0.0001).

## 4. Discussion

Mastitis is an inflammatory reaction in the bovine mammary gland that is directly related to a decrease in milk yield and a change in milk quality, and brings huge economic losses to dairy industries [1,2]. Because there are many security risks in antibiotic therapy, it is urgently necessary to find new strategies to prevent and treat bovine mastitis [17]. Studies on bovine subclinical mastitis showed that AAE could increase milk yield and reduce the somatic cell count [28]. However, there has been no report about the preventive mechanism of AAE in the LPS-induced inflammatory response of bMECs as far as we know. The results showed that CD36 participated in LPS-induced inflammation. Furthermore, AAE pretreatment effectively alleviated inflammation injury and TJP abnormality in bMECs, inhibited CD36 levels and blocked the transduction of the NF-κB pathway in response to LPS stimulation. Thus, AAE might partially act as a protective agent and prevent LPS-induced bMEC inflammatory injury to a certain extent.

The reduction in milk yield caused by mastitis is directly related to bMEC dysfunction, including the destruction of cell integrity, decreased cell viability and increased apoptosis [29]. Our study suggested that LPS had a negative effect on the ultrastructure of bMECs, such as incomplete cell membranes, fragmented nuclei, reduced mitochondrial cristae and swollen endoplasmic reticulum. Surprisingly, AAE significantly reversed the apoptosis characteristics of bMECs. Moreover, mastitis also destroys the integrity of the blood–milk barrier and disrupts the TJ by affecting the expression of TJPs [30]. Occludin, ZO-1 and claudin-1 are three important TJPs [31]. In this study, AAE could remarkably upregulate occludin, ZO-1 and claudin-1 mRNA and protein levels in bMECs injured by LPS. The morphology and barrier deterioration of bMECs are closely related to proinflammatory mediators. The bMECs can react to bacterial infection and produce inflammatory cytokines [32]. These cytokines are rapidly expressed at the initial stage of infection and are necessary for attracting leukocytes from the blood flow to bacterial invasion sites in the mammary gland [33]. However, excessive production will lead to a severe proinflammatory response and the exacerbation of tissue injury [34,35]. The results suggested that stimulation with 10 μg/mL LPS for 12 h led to the expression of IL-1β, IL-6 and TNF-α mRNA in bMECs, which elicited a cellular inflammatory response. *A. annua* extracts exert anti-inflammatory effects by inhibiting inflammatory cytokines. *A. annua* extracts could reduce the production of TNF-α and PGE2 in activated rat neutrophils challenged by LPS, and the effect of whole AAE is superior to single artemisinin at the same concentration [36]. Studies on human neuroblastoma cells also showed that the secondary metabolites in the AAE had a more obvious synergistic effect and could inhibit TNF-α mRNA expression after LPS treatment [37]. The present study indicated that AAE could significantly suppress the gene expression of IL-1β, IL-6 and TNF-α in bMECs, thereby protecting bMECs from LPS-caused inflammatory injury, TJ disruption and cell apoptosis. Interestingly, whether the anti-inflammatory effect of entire AAE on LPS-exposed bMECs is greater than that of the single active ingredient needs continued study.

The present study further explored the anti-inflammatory mechanism of AAE on the NF-κB signal transduction pathway activated by LPS. NF-κB is an important transcription factor that exists as an inactive heterodimer of p50 and p65 and binds to its inhibitor, IκB [38]. When inflammation occurs, IκB kinase (IKK) can be activated and cause phosphorylation of IκB, leading to the release and nuclear translocation of p65, and the transcription of proinflammatory factor genes is initiated [39]. LPS can result in inflammation by triggering the NF-κB signaling pathway, subsequently promoting the secretion of various inflammatory cytokines and further aggravating the inflammatory immune response during infection [40]. Our study confirmed the view that the phosphorylation levels of IκBα and p65 were significantly increased after stimulation with 10 μg/mL LPS for 12 h, resulting in the expression of TNF-α, IL-1β and IL-6. Previous studies had shown that *A. annua* extracts acted as an inhibitor of the NF-κB inflammatory pathway. In atherosclerosis-related human THP-1 monocytes, artemisinin prevented the activation of the NF-κB signaling pathway and reduced the production of proinflammatory cytokines [41]. Jiao et al. [42] also reported that *A. annua* leaves and artemisinin significantly downregulated NF-κB and IL-17α mRNA levels in the cecum of poultry infected with coccidiosis. Furthermore, artesunate could alleviate inflammation-induced liver fibrosis by inhibiting the LPS/TLR4/NF-κB signaling pathway in rats [43]. In addition, *A. annua* polysaccharides eliminated the expression of p65 in HepG2 cancer cells in a dose-dependent manner [44]. Similarly, our study indicated that AAE attenuated NF-κB signal transduction by reducing IκBα release and p65 nuclear translocation, thereby alleviating inflammation damage in bMECs stimulated by LPS.

CD36 not only affects LCFA transport but also regulates cellular immunity [45]. CD36 mediates *E. coli* recognition and proinflammatory signal transduction in response to LPS challenge, resulting in elevated IL-6 and IL-8 levels [46]. Studies showed that CD36 and TLR2 regulated ER stress-induced macrophage apoptosis [47]. In another study, intratumoral regulatory T cells in breast cancer patients exhibited increased CD36 expression; however, blocking CD36 inhibited tumor growth [48]. The current results exhibited that LPS induced a significant increase in CD36 levels in bMECs, further activating NF-κB and the release of inflammatory factors. Thus, CD36 can be a critical target to prevent the inflammatory response of bMECs. Research has indicated that Artemisia plant extracts could prevent metabolic disorders induced by a high-fat diet in mice by downregulating the expression of CD36, TNF-α, IL-6, IFN-α and IFN-β genes in epididymal adipose tissue [49]. *A. annua* extracts significantly reduced the levels of CD36 mRNA in human mesenchymal stem cells and decreased the secretion of IL-6 challenged by TNF-α [50]. In this study, AAE inhibited the LPS-induced activation of the NF-κB signaling pathway, and suppressed the expression of CD36 and inflammatory cytokines, thereby alleviating cell damage and protecting the TJ. However, whether CD36 plays a core immune-regulatory role in the protection of AAE against the LPS-induced inflammatory injury of bMECs needs to be further studied.

## 5. Conclusions

Our results suggest that AAE could alleviate the inflammatory response and protect the tight junction of LPS-induced bMECs. The anti-inflammatory mechanism was found to be partly related to the downregulation of CD36 expression and the inhibition of the NF-κB pathway. In summary, AAE has a certain protective effect on the LPS-induced inflammatory injury of bMECs.

## Figures and Tables

**Table 1:** Primer sequences of quantitative reverse transcription PCR.

**Gene Name**	**Accession** **Number**	**Primer Sequence (5′–3′) ^1^**	**Size (bp)**
TNF-α	NM_173966.3	F:CTGGCGGAGGAGGTGCTCTC	85
		R:GGAGGAAGGAGAAGAGGCTGAGG	
IL-1β	NM_174093.1	F:ATGAAGAGCTGCATCCAACACCTG	110
		R:ACCGACACCACCTGCCTGAAG	
IL-6	NM_173923.2	F:GCCTTCACTCCATTCGCTGTCTC	117
		R:AAGTAGTCTGCCTGGGGTGGTG	
CD36	NM_174010.3	F:TGCAGGTCAACATGCTGGTCAAG	126
		R:TTTCCGCCTTCTCATCACCAATGG	
Occludin	NM_001082433.2	F:GCCTGTGTTGCCTCCACTCTTG	132
		R: ACCGTAGCCATAGCCGTAGCC	
Claudin- 1	NM_001001854.2	F:TGCTGGGACTAATAGCCATCTTTGTG	83
		R:CATCTTCTGTGCCTCGTCGTCTTC	
ZO-1	XM_024982009.1	F:CCGAATGAAACCGCACACAAACC	107
		R:GTCTCCACGCCACTGTCAAACTC	
UXT	NM_001037471.2	F: AATGTCATTGAGCGACTCCAGGAAG	92
		R: GGGACCACTGTGTCAACGAAGAAG	

^1^ F = forward primer; R = reverse primer.

**Table 2:** The 30 representative active components in AAE.

**Index**	**Q1 (Da)**	**Q3 (Da)**	**Molecular Weight (Da)**	**Compound**	**Class**
mws0177	111.01	67	112.02	2-Furanoic acid	Organic acids
pme3207	141.02	59	142.03	Muconic acid	Organic acids
mws0281	191.02	111.01	192.03	Citric Acid	Organic acids
mws0470	117.02	73	118.03	Methylmalonic acid	Organic acids
mws0192	117.02	73	118.03	Succinic acid	Organic acids
pmn001578	255.23	255.23	256.22	Hexadecanoic acid	Phenolic acids
Lmlp012720	279.16	149.02	278.15	Dibutyl phthalate	Phenolic acids
Lmln010063	205.16	189.13	206.17	2,6-Di-t-butylphenol	Phenolic acids
mws0178	353.09	191.01	354.10	Chlorogenic acid	Phenolic acids
pme0281	165.02	121.03	166.03	Terephthalic acid	Phenolic acids
ML10174588	233.16	233.16	234.16	Confertifoline	Terpenoids
Hmcp003852	223.21	207.03	222.20	Elemol	Terpenoids
Lmjp006982	267.16	203.14	266.15	Deoxyartemisinin	Terpenoids
Lmjn006711	249.15	205.16	250.16	4,5-Epoxyartemisinic Acid	Terpenoids
Lmjn004991	283.16	203.14	287.18	Dihydro Artemisinin-D3	Terpenoids
pmp001309	465.1	303.1	464.10	6-Hydroxykaempferol-7-O-glucoside	Flavonoids
Lmdp003286	465.1	303.06	464.10	Isohyperoside	Flavonoids
mws0061	463.09	300	464.10	Quercetin-3-O-galactoside (Hyperin)	Flavonoids
pmb3894	329.1	229.1	330.06	Di-O-methylquercetin	Flavonoids
pme3211	463	301	464.08	Quercetin 3-O-glucoside (Isotrifoliin)	Flavonoids
pme0489	137.07	108	136.06	N-Methylnicotinamide	Alkaloids
pmp001287	120.08	103.05	119.07	N-Benzylmethylene isomethylamine	Alkaloids
pmp001275	672.42	331.29	671.41	3-Hydroxypropyl palmitate glc-glucosamine	Alkaloids
pme2268	138.05	94.07	137.05	Trigonelline	Alkaloids
pmp001198	132.1	57.1	131.10	6-Deoxyfagomine	Alkaloids
pmp000605	449.1	287.05	448.08	Rhamnone-2-O-B-D-Glucopyranoside from Italy	Quinones
pmp000608	493.13	331.08	492.13	Aurantio-obtusin-6-O-Glucoside	Quinones
pmn001492	187.1	123.1	188.04	Ayapin	Lignans and Coumarins
Lmgp003270	369.16	177.06	368.07	Scopoletin-7-O-glucuronide	Lignans and Coumarins
pmn001378	519.19	357.14	520.19	Pinoresinol-4-O-glucoside	Lignans and Coumarins

## Data Availability

The main analysis data can be obtained from the authors upon reasonable request.

## Supplementary Materials

The following supporting information can be downloaded at: https://www.mdpi.com/article/10.3390/ani12101228/s1, Figure S1: Whole Western blotting images for Figure 5; Figure S2: Whole Western blotting images for Figure 7; Figure S3: Whole Western blotting images for Figure 8.
