# Supplementary material for: Ethanol Extract of Artemisia Annua Prevents LPS-Induced Inflammation and Blood–Milk Barrier Disruption in Bovine Mammary Epithelial Cells
Source: Animals (Basel). 2022 May 10;12(10):1228. doi: 10.3390/ani12101228 (PMC9138109; doi:10.3390/ani12101228)
Supplement: Supplementary file 1 [file animals-12-01228-s001.zip › animals-1671055-supplementary.pdf]

Western Blotting Supplementary Materials

1. Tight Junction Proteins

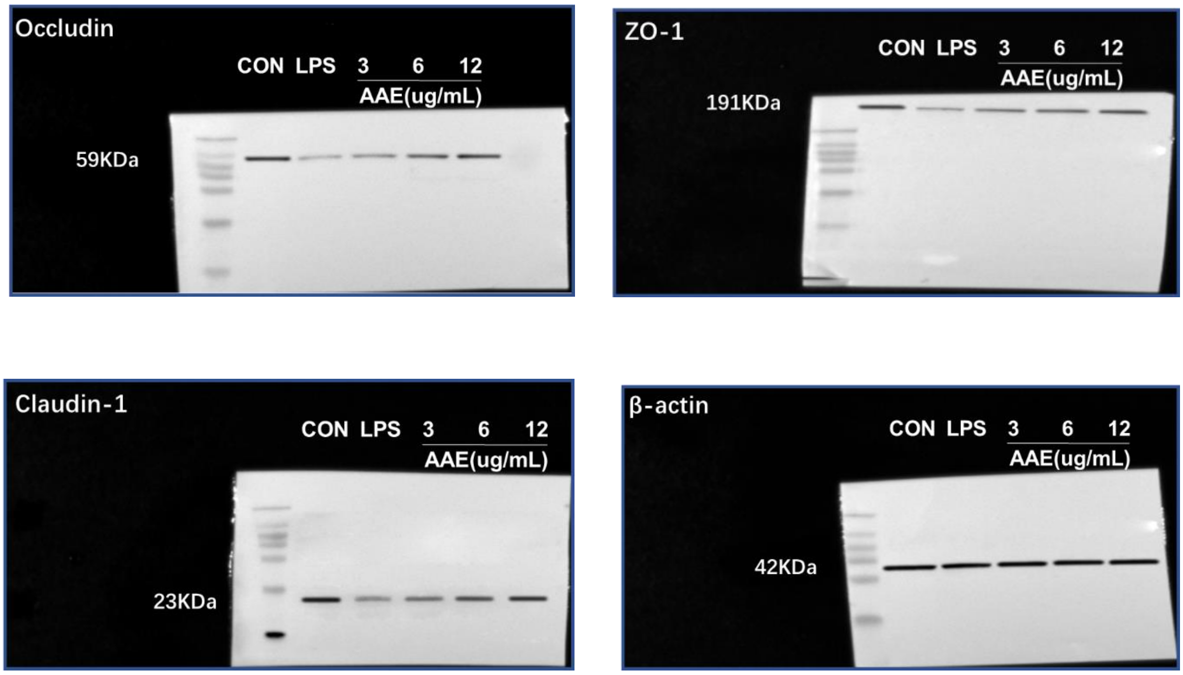

CON: cells were cultured in medium (serum-free); LPS: cells were incubated with 10 μg/mL LPS; AAE-3, AAE-6 and AAE-12: AAE (3, 6 or 12 μg/mL) + LPS (10 μg/mL).

Figure S1. Whole western blotting images for Figure 5

2. CD36

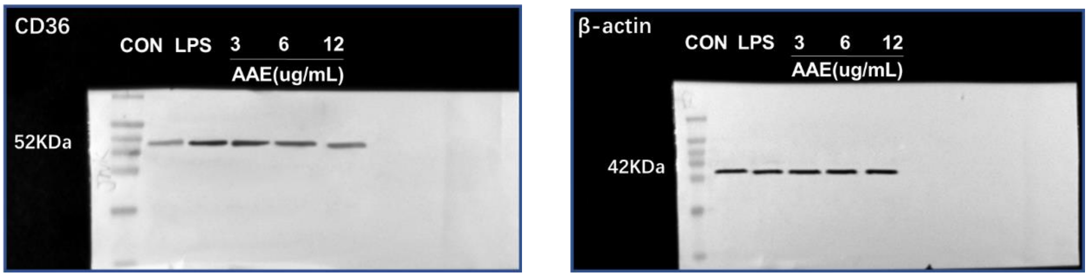

CON: cells were cultured in medium (serum-free); LPS: cells were incubated with 10 μg/mL LPS; AAE-3, AAE-6 and AAE-12: AAE (3, 6 or 12 μg/mL) + LPS (10 μg/mL).

Figure S2. Whole western blotting images for Figure 7

3. NF-κB Signaling Pathway

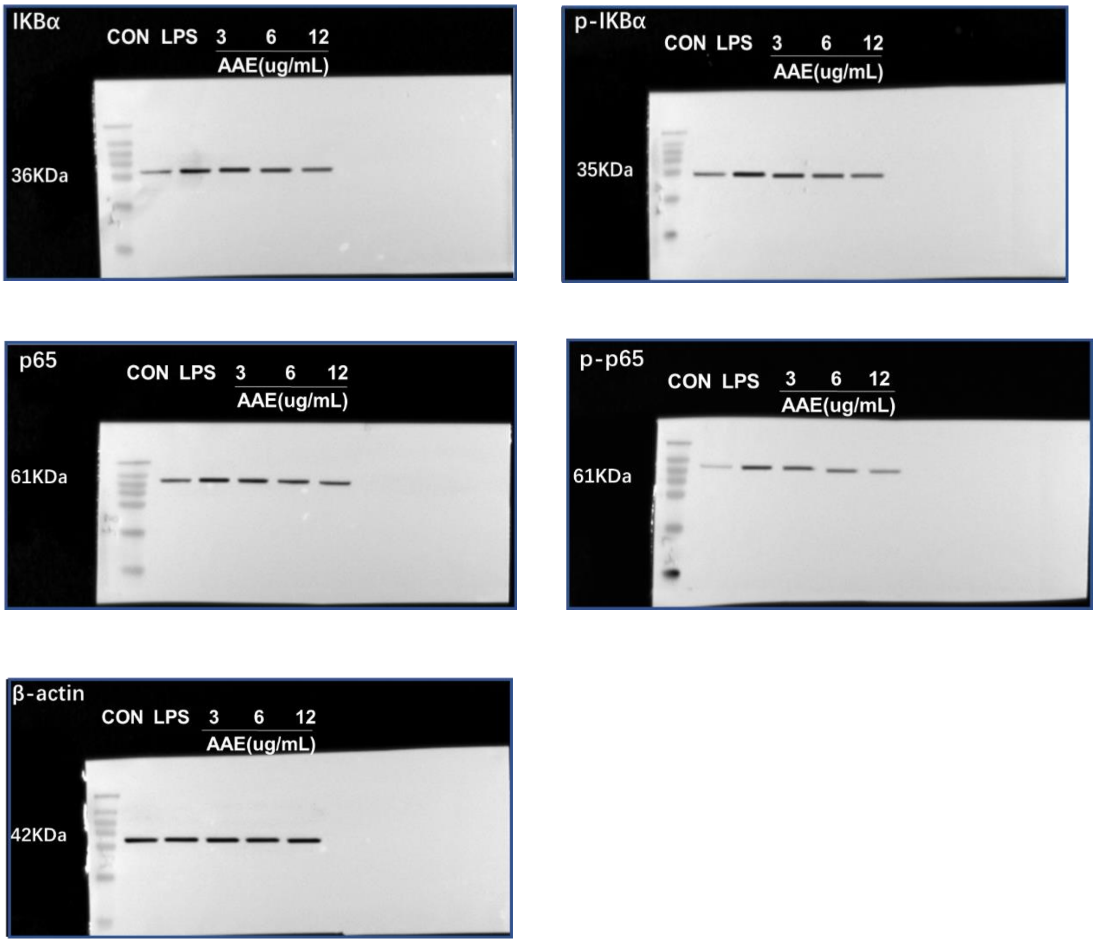

CON: cells were cultured in medium (serum-free); LPS: cells were incubated with 10 μg/mL LPS; AAE-3, AAE-6 and AAE-12: AAE (3, 6 or 12 μg/mL) + LPS (10 μg/mL).

Figure S3. Whole western blotting images for Figure 8
